# Calcium-Dependent Protein Kinase Genes in *Glycyrrhiza Uralensis* Appear to be Involved in Promoting the Biosynthesis of Glycyrrhizic Acid and Flavonoids under Salt Stress

**DOI:** 10.3390/molecules24091837

**Published:** 2019-05-13

**Authors:** Xuechen Tong, Aiping Cao, Fei Wang, Xifeng Chen, Shuangquan Xie, Haitao Shen, Xiang Jin, Hongbin Li

**Affiliations:** 1 Key Laboratory of Xinjiang Phytomedicine Resource and Utilization of Ministry of Education, College of Life Sciences, Shihezi University, Shihezi 832003, China; tongxuech@163.com (X.T.); caoaiping@shzu.edu.cn (A.C.); wangfshzu@163.com (F.W.); cxf_cc@shzu.edu.cn (X.C.); xiesq@shzu.edu.cn (S.X.); 18040833273@163.com (H.S.); 2 Ministry of Education Key Laboratory for Ecology of Tropical Islands, College of Life Sciences, Hainan Normal University, Haikou 571158, China

**Keywords:** *Glycyrrhiza uralensis*, calcium-dependent protein kinase, biosynthesis of glycyrrhizic acid and flavonoids, salt stress

## Abstract

As calcium signal sensors, calcium-dependent protein kinases (CPKs) play vital roles in stimulating the production of secondary metabolites to participate in plant development and response to environmental stress. However, investigations of the *Glycyrrhiza uralensis* *CPK* family genes and their multiple functions are rarely reported. In this study, a total of 23 *GuCPK* genes in *G. uralensis* were identified, and their phylogenetic relationships, evolutionary characteristics, gene structure, motif distribution, and promoter *cis*-acting elements were analyzed. Ten *GuCPKs* showed root-specific preferential expressions, and *GuCPKs* indicated different expression patterns under treatments of CaCl_2_ and NaCl. In addition, under 2.5 mM of CaCl_2_ and 30 mM of NaCl treatments, the diverse, induced expression of *GuCPKs* and significant accumulations of glycyrrhizic acid and flavonoids suggested the possible important function of *GuCPKs* in regulating the production of glycyrrhizic acid and flavonoids. Our results provide a genome-wide characterization of *CPK* family genes in *G. uralensis*, and serve as a foundation for understanding the potential function and regulatory mechanism of *GuCPKs* in promoting the biosynthesis of glycyrrhizic acid and flavonoids under salt stress.

## 1. Introduction

Plants have developed a series of transduction pathways to respond to various environmental stresses including salinity, light, water, temperature, and wounding [1,2,3]. Subsequent signal transduction are mediated by the second messengers in cellular and sub-cellular levels, such as enzymes, transcription factors, and cytoskeletal proteins that have evolved finally to be appropriate for physiological responses [4,5]. The calcium ion (Ca^2+^) is the ubiquitous second messenger that mediates stimuli-response coupling in the variety of physiological process and challenging environments [6,7]. Calcium-dependent protein kinases (CDPKs or CPKs) are important Ca^2+^ sensors [1,8,9], and contain four functional domains, including highly variable dual leucine-zipper kinase (DLK) motif (*N*-terminal domain) that consists of *N*-myristoylation and *S-*palmitoylation sites for sub-cellular localization, serine (Ser)/threonine (Thr) kinase catalytic domain for the direct modulation of downstream genes in Ca^2+^-mediated networks [10], the autoinhibitory junction domain as an autoinhibitor for CPK inactivation or activation through the Ca^2+^ signal [11,12,13], and the calmodulin-like domain for coordinating the Ca^2+^ [3,14]. Compared to the other Ca^2+^ sensors, CPKs contain two unique domains—the Ser/Thr kinase domain and effective factor (EF)-hand calmodulin-like domain—that endow their function to phosphorylate downstream genes [14]. 

CPKs are involved in the response to a broad variety of abiotic and biotic stresses. In *Arabidopsis*, *AtCPK10* can interact with *AtHSP1* to regulate Ca^2+^-mediated stomata movement in response to drought [1,15]. The mutants of *AtCPK4* and *AtCPK11* enhance the tolerance to salt and drought stresses [16]. The overexpression of *AtCPK6* and *AtCPK23* decreases the plant tolerance to salt and drought stresses [17,18]; *AtCPK4*, *AtCPK11* [16,19], and *AtCPK23* [17] mediate plant salt stress by controlling K^+^ channels. In *Oryza sativa*, transgenic plants overexpressing *OsCPK7* or *OsCPK13* have been proved as positive regulators of resistance to salt, cold, and drought stresses [1,20]; *OsCPK21* responds to salt stress by increasing the expression of high salinity-inducible genes in transgenic *Oryza* [1,21]. Tobacco *NtCPK2* interacts with mitogen-activated protein kinases (MAPKs) to regulate the resistant response to abiotic and biotic stresses [1,22]. Additionally, *CPK* gene family members have been shown to be involved in plant growth and development [23,24]. *AtCPK17/34* controls the pollen tube tip elongation [11,23]. *OsCPK9* has been found to positively regulate the pollen viability, thus increasing spikelet fertility in rice [25]. The overexpression of *OsCPK2* strongly disrupts seed development at an early stage in transgenic rice [11,26]. 

*Glycyrrhiza* is an important medicinal plant, for its roots are used as the raw material to extract pharmacodynamic metabolites such as glycyrrhizic acid and flavonoids; it is mainly distributed in the northwest arid, semi-arid, and desert regions of China [27,28]. *G. uralensis* is the main species of *Glycyrrhiza* [29], which is also considered as an effective plant to reduce the salt content, promote organic substance, and improve the water retention ability of saline soils [28,30]. Furthermore, *G. uralensis* can be used as natural spice and food sweeteners in the food industry, and is an important medicinal product because of its abundant pharmacological activities and values [31,32]. In the extractions of *G. uralensis* roots, glycyrrhizic acid and flavonoids are the major medicinal substances for the therapy of disease [33]. Currently, the *G. uralensis CPK* gene family members and their potential function in the biosynthesis of glycyrrhizic acid and flavonoids are largely unknown. In this study, we performed a genome-wide investigation and expression profiling of the *CPK* gene family in *G. uralensis*. A total of 23 *GuCPK* genes were identified, and the comprehensive analysis of chromosomal location, phylogenetic relationship, gene structure, and motif distribution were obtained. *Cis*-element distribution and expression pattern analyses of tissue-specificity and the response to CaCl_2_ and NaCl suggested that *GuCPKs* have diverse functions. Particularly, expression analysis coupled with the determination of glycyrrhizic acid and flavonoids under treatments of 2.5 mM of CaCl_2_ and 30 mM of NaCl revealed that *GuCPK* may perform an important function in controlling the production of glycyrrhizic acid and flavonoids. These results provide systematic evolutionary and functional information on the *GuCPK* gene family, and establish the foundation for further understanding of the *GuCPKs* roles in promoting the biosynthesis of medicinal ingredients of glycyrrhizic acid and flavonoids. 

## 2. Results

### 2.1. Identification of CPK Family Genes in G. uralensis 

**Figure 1:**
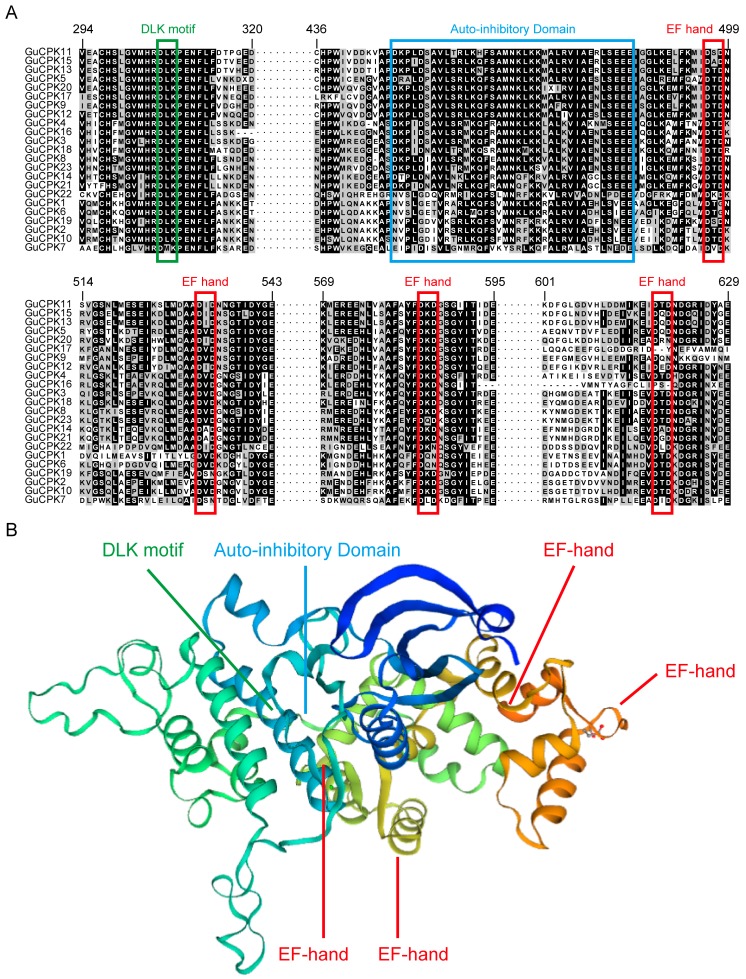
Multiple sequence alignment and conserved domain analysis of the *G. uralensis* calcium-dependent protein kinases (GuCPKs). (**A**) Sequence alignment and conserved domain analysis of the 23 GuCPKs. The detailed protein sequence information of the 23 GuCPKs is provided in Supplementary Table S3. The partial GuCPK protein sequences were selected and the corresponding conserved domains were boxed by different colors. (**B**) Three-dimensional structure of the GuCPK conserved domains. The structure was created using the Swiss-Model workspace (http://swissmodel.expasy.org) for GuCPK12 as a representative sequence. The regions of the conserved domains are indicated.

**Figure 2:**
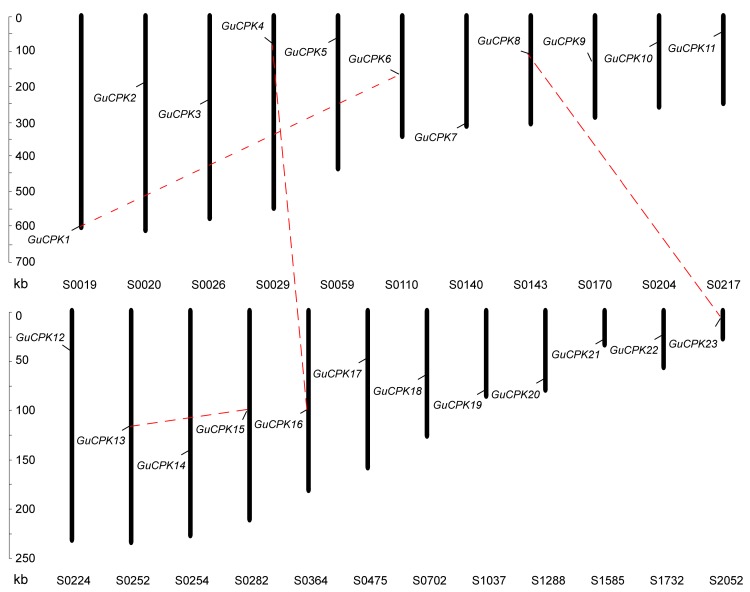
Scaffold distribution of *GuCPKs* in *G. uralensis*. *GuCPK* genes are located on 23 individual scaffolds. Vertical columns represent the scaffolds with scaffold numbers at the bottom and scale bars on the left. Pairs of segmentally duplicated genes are indicated by red dashed lines.

CPK protein sequences of *Arabidopsis* and *Glycine max* were provided as direct queries against the *G. uralensis* genome to perform BLASTP and hidden Markov Model (HMM) to identify the *GuCPK* gene family members. A total of 23 *GuCPK* genes were identified; hereinto, eight sequences of *GuCPK2*, *GuCPK6*, *GuCPK9*, *GuCPK10*, *GuCPK13*, *GuCPK16*, *GuCPK17,* and *GuCPK22* showed spliced error as the incomplete assembly of the *G. uralensis* genome. According to the eight partial *GuCPK* seuqences, we designed gene-specific primers (Supplementary Table S1) to obtain the full-length open reading frame (ORF) by polymerase chain reaction (PCR). All the 23 *GuCPK* ORF sequences were provided in Supplementary Table S2. The *GuCPK* ORFs ranged from 1482 to 2097 bp, and the encoded proteins of amino acids (aas) were in the range of 493 to 698 aa with molecular weight (MW) from 55.77 to 78.45 kDa, and the isoelectric points (*p*I) were from 5.18 to 9.22, and of the subcellular location predictions to the plasma membrane (Supplementary Table S3). Multiple sequence alignment analysis indicated that all of the 23 GuCPKs had high conserved domains, including a DLK motif, an autoinhibitory domain, and four Ca^2+^-binding EF-hand motifs (Figure 1A), and a three-dimensional structure of these domains was performed to highlight the corresponding regions (Figure 1B). Our bioinformatic analysis revealed that 17 GuCPK proteins (the 23 GuCPKs except GuCPK8, GuCPK11, GuCPK13, GuCPK15, GuCPK18, and GuCPK20) harbored potential *N*-myristoylation sites, and 12 GuCPK proteins (GuCPK3, GuCPK4, GuCPK6, GuCPK7, GuCPK8, GuCPK10, GuCPK14, GuCPK16, GuCPK18, GuCPK21, GuCPK22, and GuCPK23) contained *S*-palmitoylation sites (Supplementary Table S3). For genomic location analysis, the 23 *GuCPK* genes were anchored onto 23 different scaffolds in the *G. uralensis* genome; four paralogous gene pairs (*GuCPK1*/*GuCPK6*, *GuCPK4*/*GuCPK16*, *GuCPK8*/*GuCPK23*, and *GuCPK13*/*GuCPK15*) were found to be segmentally duplicated (Figure 2). 

### 2.2. Phylogenetic Relationship, Gene Structure, and Motif Distribution Analyses of GuCPK Genes

**Figure 3:**
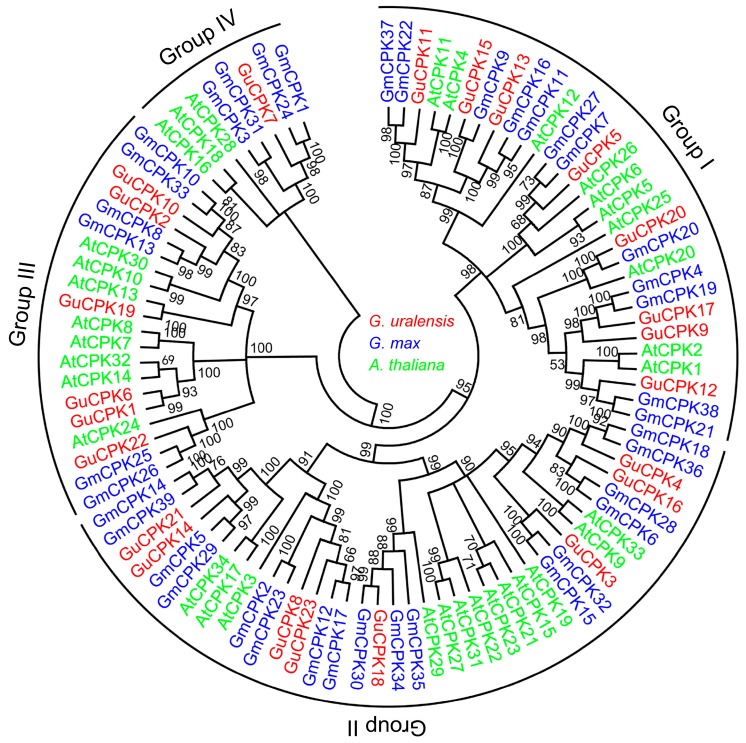
Phylogenetic relationship analysis of the CPK family. A total of 96 CPK proteins from *G. uralensis* (GuCPK), *Arabidopsis* (AtCPK) and *G. max* (GmCPK) were used to constructed a phylogenetic tree by ClustalX 2.0 and MEGA5.0 software with the neighbor-joining (NJ) method of 1000 bootstrap replicates. The CPK proteins of *G. uralensis*, *Arabidopsis*, and *G. max* were shown in red, green, and blue, respectively.

**Figure 4:**
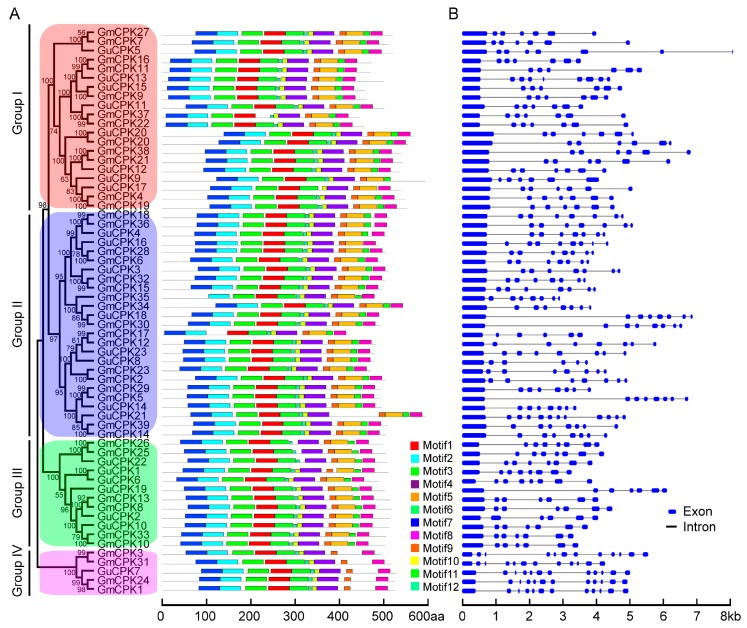
Motif distribution and exon/intron structure analyses of *CPK* family genes in *G. uralensis* and *G. max*. (**A**) Conserved motif analysis of CPK proteins. The GuCPK and GmCPK protein sequences were used to identify conserved motifs by the MEME program. (**B**) Exon/intron structure analysis of *CPK* genes. Blue boxes and black lines represent the exons and introns respectively, and the genomic length is indicated at the bottom.

In order to investigate the phylogenetic relationships of *GuCPK* gene family members, a total of 96 CPK proteins from *G. uralensis*, *Arabidopsis*, and *G. max* were used to construct the phylogenetic tree, which indicated that the CPKs were clustered into four major groups (groups I–IV) (Figure 3). The GuCPK proteins had 12 conserved motifs distributing on different positions (Figure 4A). The exon/intron organization analysis indicated that the *GuCPK* members of different groups showed diverse gene structures (Figure 4B). This demonstrated that *GuCPKs* in the same group had highly conserved motif distributions and similar exon/intron structures. 

### 2.3. Duplication Events and Syntenic Analyses of GuCPK Family Gene Members

**Figure 5:**
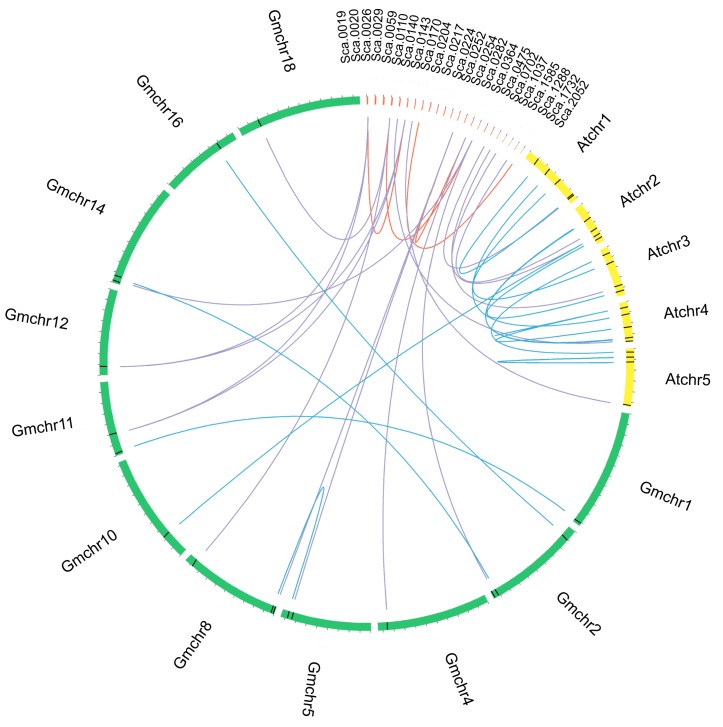
Segmental duplication of *GuCPK* genes and syntenic analysis of *G. uralensis*, *G. max,* and *Arabidopsis CPK* genes. Chromosomes and scaffolds were shown in different colors with circular form. The positions of the *CPK* genes were marked with black lines on the circle. The duplicated *CPK* pairs in *G. uralensis* were linked by red lines, syntenic relationships between *G. uralensis* and the other two species were linked by purple lines, and blue lines indicated the *CPK* pairs between or inside *G. max* and *Arabidopsis*.

To investigate the expansion of the *CPK* gene family in *G. uralensis*, syntenic analysis of *G. uralensis*, *Arabidopsis,* and *G. max* was performed using the Circos software to visualize the results. Four segmental duplication events were identified, and the paralogue gene pairs were defined as the syntenic genes (*GuCPK1/GuCPK6*, *GuCPK4/GuCPK16*, *GuCPK8/GuCPK23*, and *GuCPK13/GuCPK15*), while no tandem duplication events were discovered as the incomplete assembly of the *G. uralensis* genome (Figure 5). The *Ka*/*Ks* ratio was used to evaluate the selection pressure for the duplicated *GuCPK* paralogue gene pairs. The results of the *Ka*/*Ks* ratios of the duplicated *GuCPKs* indicated that they have undergone purifying selection (Table 1). Meanwhile, the evolutionary rates of these duplication pairs were assessed by calculating the Tajima relative rate. The results showed that *GuCPK4/GuCPK16* and *GuCPK13/GuCPK15* had prominently accelerated evolutionary rates (Table 2). 

### 2.4. Analysis of Cis-Acting Elements in the Promoter Region of GuCPK Genes

**Figure 6:**
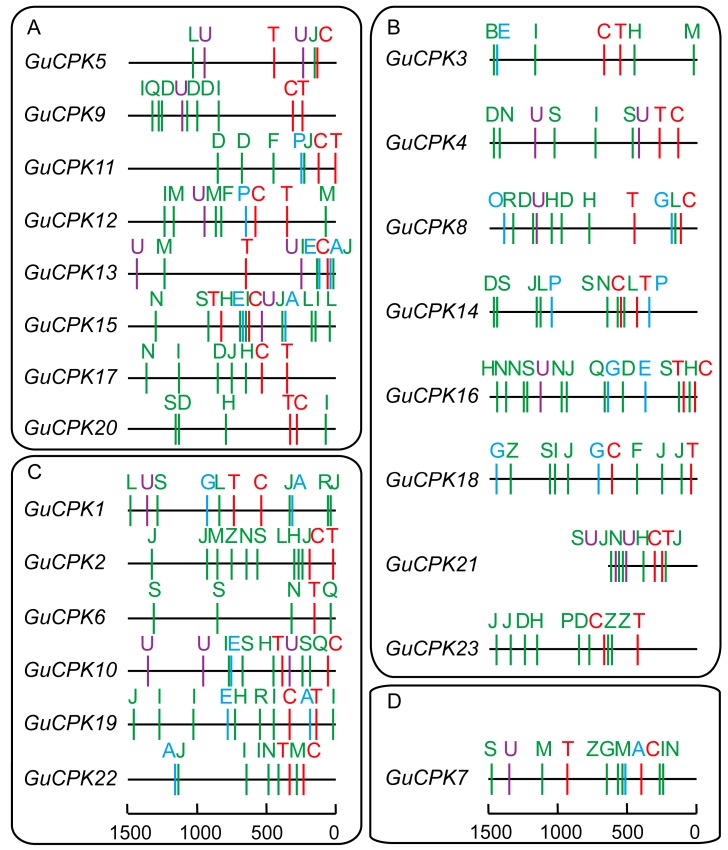
Distribution of *cis*-elements in *GuCPK* gene promoter regions. The *cis*-elements of the 1.5-kb upstream promoter regions of *GuCPKs* were predicted using PlantCARE software. The *GuCPKs* were divided into four groups according to the phylogenetic subfamilies (groups I–IV). Colored capital letters represent different *cis*-elements at corresponding positions. Red, green, purple, and blue letters indicate core elements, phytohormone response, stress response, and the high transcription of *cis*-elements, respectively. Detailed information for each *cis*-element is provided in Supplementary Table S4.

To investigate the regulatory feature of *GuCPK* genes, the isolated 1.5-kb sequence upstream of the translational start site of the *GuCPKs* was selected to analyze the constitutions of *cis*-acting elements. Besides the core elements of the putative TATA box and CAAT box, the *GuCPK* promoters mainly showed the regulatory elements of phytohormone responsiveness and stress responsiveness (Figure 6, Supplementary Table S4). The promoters of *GuCPK1*, *GuCPK13*, *GuCPK15*, *GuCPK17*, *GuCPK19*, and *GuCPK22* contained an ACGT-containing abscisic acid (ABA) response element (ABRE) involving in the ABA responsiveness, suggesting potential regulation in the ABA-signaling pathway. The ethylene-responsive element (ERE) was detected in the promoter regions of *GuCPK3*, *GuCPK10*, *GuCPK13*, *GuCPK15*, *GuCPK16*, and *GuCPK19*, indicating the function of these genes in the ethylene-signaling pathway. Additionally, the gibberellin (GA) responsive elements (GARE and P-box) were found in seven *GuCPK* promoters. One or two v-myb avian myeloblastosis viral oncogene homolog (MYB) binding sites involved in flavonoid biosynthetic gene regulation elements (MBSI) were present in more than half of *GuCPK* promoters, providing a potential regulation function of *GuCPK* genes in the flavonoid biosynthetic pathway. TC-rich elements that participated in defense and stress responsiveness were discovered in nine *GuCPK* promoters that were classified in Cluster I and Cluster II. Moreover, the elements of anaerobic induction (ARE) and low-temperature responsiveness (LTR) were also observed in five *GuCPK* promoters. Several light-responsive elements, including mineralocorticoid response element (MRE), I-box, G-box, Box-I, AE-box, and 3-AF1 were shown in nearly all *GuCPK* promoters. These results demonstrated that *GuCPK* genes might perform diverse functions to regulate plant development and respond to environmental stresses through their involvement in hormonal signal transduction. 

### 2.5. Tissue-Specific Expression Pattern Analysis of GuCPKs

**Figure 7:**
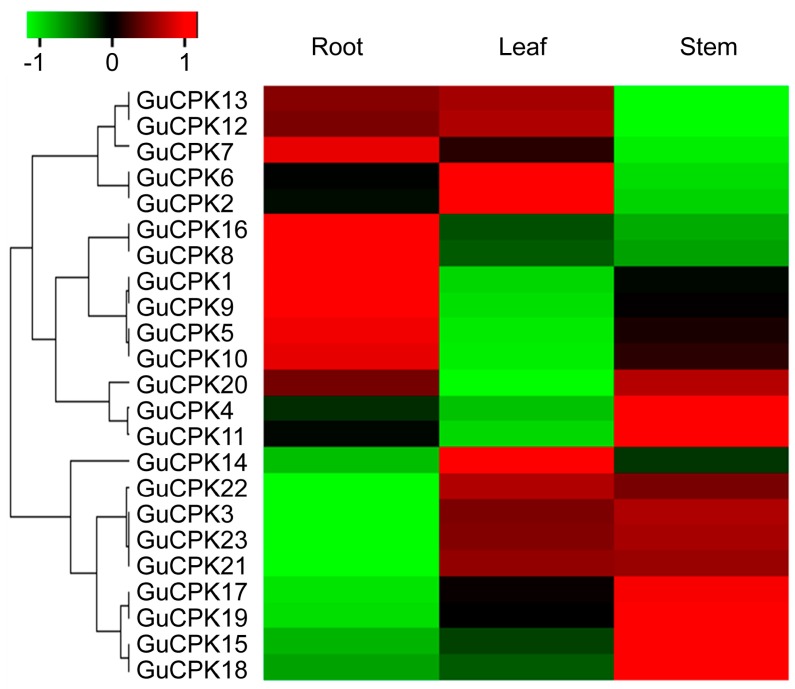
Heatmap of expression levels of *G. uralensis GuCPKs* in different tissues. The expression levels of *GuCPKs* were determined by qRT-PCR. The *GuCPK* genes were ordered by the cluster of their expression patterns. Each value denotes the mean relative level of expression of three replicates. Higher or lower expressions of *GuCPK* genes in different tissues were colored by red or green, respectively. The color bar is shown in the upper apex. The visualized heatmap was generated using R package based on the qRT-PCR data.

Tissue-specific expression patterns of *GuCPKs* in different *G. uralensis* tissues were performed by quantitative real-time polymerase chain reaction (qRT-PCR) (Figure 7), with the gene-specific primers listed in Supplementary Table S1. The results that were visualized by qRT-PCR based heatmap showed that *GuCPKs* exhibited diverse tissue-specific expression features. *GuCPK16* and *GuCPK8* displayed the most accumulated expressions in roots, and *GuCPK1, GuCPK9, GuCPK5,* and *GuCPK10* indicated higher expression in roots than in leaves and stems, implying their potential important functions in root development and related physiological metabolic process. *GuCPK14*, *GuCPK6*, and *GuCPK2* demonstrated increased expressions in leaves, suggesting their possible role in leaf developmental and physiological process. In terms of the expressions in stems, *GuCPK17, GuCPK9, GuCPK15, GuCPK8,* as well as *GuCPK1* and *GuCPK11* indicated specific or dominant expressions. *GuCPK13, GuCPK12, GuCPK7, GuCPK6,* and *GuCPK2* were enriched in roots and leaves than in stems. *GuCPK22, GuCPK3, GuCPK23,* and *GuCPK21* were preferentially expressed in leaves and stems than in roots (Figure 7). Furthermore, the expression patterns of the *GuCPK* duplicated paralogous gene pairs were analyzed. The four duplication pairs had diverse expression profiles in different tissues, suggesting the four paralogous gene pairs shared their potential functional divergence after duplication events.

### 2.6. Expression Profile of GuCPKs in Response to CaCl_2_ and NaCl Treatments

**Figure 8:**
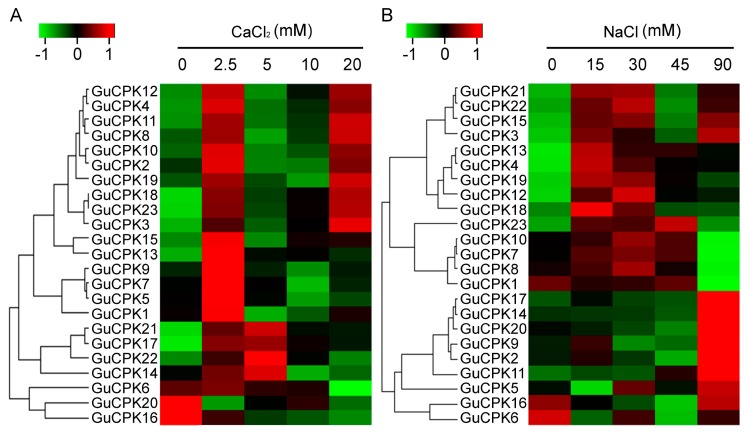
Heatmap of the expression levels of the 23 *GuCPK* genes in *G. uralensis* roots under different concentrations of CaCl_2_ and NaCl treatments. (**A**) Heatmap of 23 *GuCPKs* under 0 mM, 2.5 mM, 5 mM, 10 mM, and 20 mM of CaCl_2_ stimulations. (**B**) Heatmap of *GuCPKs* under 0 mM, 15 mM, 30 mM, 45 mM, and 90 mM of NaCl treatments. Each value is the mean of three independent replicates. *GuCPK g*enes that were highly or weakly expressed in different treated materials were colored by red or green, respectively. The color bar was indicated in the upper apex. The visualized heatmap was generated using R package on the basis of the qRT-PCR data.

In the light of CPKs’ important roles in calcium signal transduction and in response to salt stress [34], in order to analyze the *GuCPK* expression profiles under CaCl_2_ and NaCl treatments, the relative expression levels of the 23 *GuCPKs* were detected and visualized by a qRT-PCR based heatmap using the materials treated by CaCl_2_ and NaCl with different concentrations. The results showed that almost all the *GuCPKs* demonstrated a prompt upregulation after 2.5 mM of CaCl_2_ treatment, except for *GuCPK20*, which showed a significant decrease expression under any concentration point of CaCl_2_ treatment. In addition, there were four (*GuCPK21, GuCPK17, GuCPK22,* and *GuCPK14*) and 10 *GuCPKs* (*GuCPK12, GuCPK4, GuCPK11, GuCPK8, GuCPK10, GuCPK2, GuCPK19, GuCPK18, GuCPK23,* and *GuCPK3*) that indicated significant accumulations after 2.5 mM and 20 mM of CaCl_2_ treatment, respectively. Three *GuCPKs* (*GuCPK6, GuCPK20,* and *GuCPK16*) displayed decreased expressions after the supplement of CaCl_2_ (Figure 8A). In terms of NaCl treatment, the expressions of the 23 *GuCPKs* demonstrated two typical clusters; one contained 14 *GuCPKs* that showed accumulated expressions after 15 mM or 30 mM of NaCl treatment, and another included the remaining *GuCPKs* (except *GuCPK6*) that indicated significant enrichments after 90 mM of NaCl stimulation (Figure 8B).

### 2.7. Correlation Analysis of GuCPKs Expression and Biosynthesis of Glycyrrhizic Acid and Flavonoids of G. Uralensis under CaCl_2_ and NaCl Treatments

**Figure 9:**
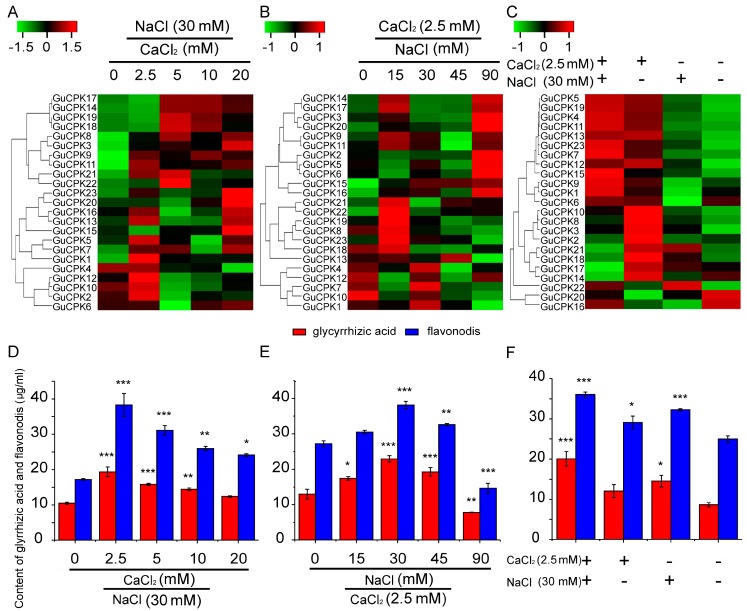
Expression profiles of the *GuCPK* genes and contents of the glycyrrhizic acid and flavonodis in *G. uralensis* roots treated by different concentration of CaCl_2_ and NaCl. (**A**) Heatmap of *GuCPKs* expressions at 30 mM of NaCl and different concentration CaCl_2_ stimulations. (**B**) Heatmap of *GuCPKs* expressions at 2.5 mM of CaCl_2_ and different concentration NaCl stimulations. (**C**) Heatmap of *GuCPKs* expressions at different formulations of with or without treatments of 2.5 mM of CaCl_2_ and 30 of mM NaCl. Each value denotes the mean relative level of the expression of three independent replicates. *GuCPK* genes with higher or lower expressions in different treated tissues were colored by red or green, respectively. The color bars are shown at the upper apex. The qRT-PCR-based heatmap was visualized by R package. The contents of glycyrrhizic acid and flavonoids were measured under the treatments of 30 mM of NaCl and different concentrations of CaCl_2_ (**D**), and 2.5 mM of CaCl_2_ and different concentrations of NaCl (**E**), as well as different formulations with or without 2.5 mM of CaCl_2_ and 30 mM of NaCl (**F**). Red and blue represent the contents of glycyrrhizic acid and flavonoids, respectively. Each value represents the mean relative level of the expression of three independent experiments. The data are indicated as mean values ± SD. Asterisks indicate significant differences based on Student’s *t*-test using the materials without any addition of CaCl_2_ or NaCl as controls, * *p* < 0.05; ** *p* < 0.01; *** *p* < 0.001.

Considering that the promotions of secondary metabolites by salt stress were observed, and that glycyrrhizic acid and flavonoids were the main medicinal ingredients in *G. uralensis* [31,32], therefore, the appropriate concentrations of CaCl_2_ and NaCl were chosen to treat the *G. uralensis* roots to investigate the correlation between *GuCPK* expression and the biosynthesis of glycyrrhizic acid and flavonoids. The *GuCPKs* exhibited predominant expressions and rapid responsiveness under treatments of 2.5 mM of CaCl_2_ and 30 mM of NaCl (Figure 8, Supplementary Figure S1). In addition, after the supplement of 30 mM of NaCl and different concentrations of CaCl_2_, the *GuCPKs* indicated distinct expression patterns, and the content of both the glycyrrhizic acid and flavonoids reached the peak value at the point of 2.5 mM of CaCl_2_ (Figure 9A,D). When 2.5 mM of CaCl_2_ was appended, after the addition of different concentrations of NaCl, the *GuCPKs* showed diverse expression features, and the content of both the glycyrrhizic acid and flavonoids accumulated the highest level at the concentration of 30 mM of NaCl (Figure 9B,E). Interestingly, by the application of the mixture of 2.5 mM of CaCl_2_ and 30 mM of NaCl, the *GuCPKs* displayed an obvious expression pattern with the 12 *GuCPKs* that exhibited significant increased expression. Meanwhile, the content of glycyrrhizic acid and flavonoids were also measured to be the maximum value (Figure 9 C,F). These results suggest that there may exist a close link between the significant accumulation of *GuCPKs* and the promoted production of glycyrrhizic acid and flavonoids under salt stress.

## 3. Discussion

*CPK* gene family members play important roles in many aspects of plant development and particularly in the involvement of stress-induced secondary metabolite production [8], and have been systematically investigated in some plants, including 34 members of *Arabidopsis*, 39 members of *G. max*, 31 members of *O. sativa*, 40 members of *Z. mays*, and 30 members of *H. brasiliensis* [3,24,35,36]. In this study, through a genome-wide analysis, we identified 23 *GuCPK* gene family members that were anchored onto different scaffolds of the *G. uralensis* genome (Supplementary Table S3, Figure 2). Due to the limitation of the *G. uralensis* genome that leads to eight incomplete sequences of *GuCPKs*, we obtained the full-length ORF of the eight *GuCPKs* by PCR-based sequence amplifying (Supplementary Table S2, Figure 1).

Plant CPKs share typical domains, which endow the CPK enzymatic activity to phosphorylate the substrate and accordingly to transduce the cellular calcium signal [12]. GuCPKs have the similar conserved domains that contain the DLK motif, autoinhibitory domain, and Ca^2+^-binding EF-hand motifs (Figure 1). The DLK motif is highly variable and includes *N*-myristoylation and *S*-palmitoylation sites for sub-cellular localization [19,28]; 21 AtCPKs have two of those sites that decide their localization on the plasma membrane in *Arabidopsis* [19,37]. ZmCPK1 had a *N-*myristoylation site and was predominately localized to the cytoplasm and nucleus [38]. A total of 17 and 12 of the GuCPK proteins contained potential *N*-myristoylation and *S*-palmitoylation sites, respectively. The prediction of the subcellular distribution of the 23 GuCPK proteins are localized to the plasma membrane (Supplementary Table S3), indicating that the *N*-myristoylation and *S*-palmitoylation sites of GuCPK might be functional in membrane targeting and the stability of GuCPK proteins. The number and position of EF-hands had critical roles in activating the CPK by Ca^2+^-binding affinities [39,40]. All the GuCPKs contained four EF-hands, which were also found in *Arabidopsis*, rice, and maize, allowing the CPK proteins as a Ca^2+^ sensor to fulfill the potentially similar biological functions [11].

The expansion and divergence of the *CPK* gene family could be estimated by the conservation and diversity of the gene structure [19]. GuCPKs were clustered into four major groups (Figure 3). Group IV expanded the first from the evolutionary branch, suggesting that it has the longest evolutionary history [41]. Group I and Group II originated from Group III in the evolutionary history. All the GuCPKs have similar motif distributions, and the *GuCPK* members in the same or different group have similar or distinct gene structures (Figure 4). Gene family expansion has two main mechanisms: segmental and tandem duplications [24,42]. In this study, no tandem duplication was discovered that contributed to the expansion of the *G. uralensis CPK* family (Figure 5), which may be caused by the incomplete assembly of the *G. uralensis* genome. The *Ka*/*Ks* ratios of the four duplicated *GuCPK* paralogue gene pairs (Table 1) indicated a functional constraint with purifying selection on the *GuCPK* genes [38,43]. Four segmental duplications were identified in the *G. uralensis* genome. These four duplicated gene pairs shared similar motif distributions (Figure 4), while it is observed that the two *GuCPK* genes of the same gene pair have different gene structures and diverse *cis*-acting element constitutions (*GuCPK8/GuCPK23* and *GuCPK13/GuCPK15*) (Figure 4 and Figure 6), suggesting that the potential functional similarity or divergence of the four duplicated gene pairs may occur after duplication events, showing the consistence with the results in *H. brasiliensis* [3].

Commonly, the similarity of the sequences of *CPK* family gene members might decide their phylogenetic classifications and possible identical functions. In this study, *AtCPK4* and *AtCPK11,* together with *GuCPK11*, *GuCPK13,* and *GuCPK15,* were classified into Group Ι (Figure 3) with a showing of highly sequence similarity, which have been proven to be involved in the response to abiotic stresses of salt and drought [16], ABA signal transduction, and ROS response [11]. The duplicated gene pair *GuCPK13* and *GuCPK15* showed similar expression patterns under different treatments (Figure 8 and Figure 9), implying their likely same functions in response to environmental stress. The duplicated gene pair of *GuCPK1*/*GuCPK6* exhibited higher similarity with *AtCPK23* that was an opposite regulator in abiotic stresses [18,44] and indicated the ABA sensitivity [45]. *GuCPK7*, the only *G. uralensis CPK* member in Group IV, indicated significant accumulation after the stimulation of the mixture of 2.5 mM of CaCl_2_ and 30 mM of NaCl (Figure 8 and Figure 9), and shared high identity with *AtCPK28* that was a negative regulator of Ca^2+^ burst [45].

CPKs perform important roles as calcium sensors to participate in hormone signaling [46]. In *Arabidopsis*, AtCPK4 and AtCPK11 phosphorylate two ABA-responsive transcription factors of ABF1 and ABF4 to positively influence the ABA-reduced seed germination, stomatal movement, and seedling growth [16]; AtCPK32 can directly phosphorylate ABF4 in vitro, and the overexpression of *AtCPK32* can enhance ABA sensitivity and the expression of ABF4-regulated genes in transgenic *Arabidopsis* [37]. *AtCPK10* plays an essential role in the ABA-mediated and Ca^2+^-mediated regulation of stomatal closure [47]. *OsCPK1* has been found as a GA biosynthesis negative regulator that refines the endogenous GA concentration and reduces drought stress injury in rice [48]. Tomato *LeCPK2* controls the ethylene production to modulate the wounding signaling by phosphorylating *LeACS2* [11,49]. The expressions of *HbCPKs* in *H. brasiliensis* were significantly induced by ethylene stimulation [3]. The promoter regions of *G. uralensis GuCPKs* contained many hormone responsiveness elements of ABA, ethylene, and GA (Figure 6, Supplementary Table S4), which provides the possibility that the *GuCPKs* expressions are under the control of these phytohormones.

Plant *CPKs* have demonstrated the critical roles in response to abiotic stresses [45]. The expressions of *CPK* genes are reported to indicate tissue-specific features that are involved in plant organ development and secondary metabolite generation, and are regulated by various environmental stimuli. Studies have shown that *ZmCPK37* is specifically expressed in root, and the transcript level increased under salt treatment in *Z. mays* [24]. *HbCPK12* and *HbCPK28* were involved in rubber latex production upon ethylene stimulation in *H. brasiliensis* [3]. In this study, *GuCPKs* exhibited rapid upregulation to the stimulations of CaCl_2_ and NaCl, or both, with the most significant enriched expressions after the co-supplementation of 2.5 mM of CaCl_2_ and 30 mM of NaCl (Figure 8 and Figure 9, Supplementary Figure S1), showing similar *CPK* expression patterns under the treatment of salt stress in *S. lycopersicum* [15], which supports the notion that *GuCPKs* are central regulators in the responsiveness of the early stage of salt stress.

Generally, wild *G. uralensis* is a salt-tolerant medicinal legume, which can be utilized for the bioremediation of salt-affected soils [50]. The salt tolerance of *G. uralensis* is increased by the plant growth-promoting rhizobacteria to improve shoot nitrogen content [51]. It is also reported that moderate salt stimulation promotes the production of secondary metabolites such as rographolide, deoxyandrographolide, dehydroandrographolide, and total lactones in *Andrographis paniculata,* and essential oil in *Salvia sclarea* [52,53]. The contents of glycyrrhizic acid and flavonoids were significantly promoted after appropriate stimulations of 2.5 mM of CaCl_2_ and 30 mM of NaCl, and reached the peak value by supplementation of the mixture of 2.5 mM of CaCl_2_ and 30 mM of NaCl (Figure 9), suggesting that moderate environmental stress may be a positive regulator to produce glycyrrhizic acid and flavonoids. Glycyrrhizic acid can act as the scavenger to eliminate the reactive oxygen species (ROS)-induced generation of free radicals under salt and drought stresses [54,55,56]. The production of glycyrrhizic acid and liquiritin were increased significantly in *G. uralensis* roots under drought stress [57]. On the basis of the above, and given that the significant specific accumulation in roots and the prompt induced expression of *GuCPKs* under salt stress, it can be inferred that there may be a close correlation between the upregulated expression of *GuCPKs* and the biosynthesis of glycyrrhizic acid and flavonoids in *G. uralensis* under salt stress.

## 4. Materials and Methods

### 4.1. Genome-Wide Identification of GuCPK Family Genes

*Arabidopsis* and *G. max* CPK protein sequences were used to query the *G. uralensis* genome using BLASTP [25]. All the putative GuCPK candidates were manually verified with the InterProScan program (http://www.ebi.ac.uk/interpro/) to assess the presence of the protein kinase domain (PF00069) and EF-hand_7 domain (PF13499). A careful manual review of these sequences was carried out to amend the underlying mistake of the *G. uralensis* genome database. A total of eight *GuCPKs*—*GuCPK2*, *GuCPK6*, *GuCPK9*, *GuCPK10*, *GuCPK13*, *GuCPK16*, *GuCPK17*, and *GuCPK22*—were misaligned sequences. The misaligned sequences and deleted loci were mainly verified. The full-length primers were designed and used to obtain the correct sequence by PCR assay.

### 4.2. Analyses of Phylogenetic Relationship and Gene Structural Organization

*GuCPK*s were mapped to different scaffolds by identifying the genomic location on the basis of the *G. uralensis* genome database. The amino acid sequences of CPKs in *G. uralensis*, *Arabidopsis,* and *G. max* were used to build a phylogenetic tree according to the neighbor-joining method with 1000 bootstrap tests using the MEGA5.0 program (Hachioji, Tokyo, Japan). Multiple sequence alignments were conducted using Clustal X2.0 (Belfield, Dublin, Ireland). The conserved motif analysis of GuCPK was performed by Multiple Em for Motif Elicitation (MEME) online software (http://meme-suite.org/tools/meme). The relative molecular weight and *p*I of GuCPKs were predicted by the ExPASy server (http://expasy.org/). The sub-cellular location of GuCPKs was predicted by Softberry (http://linux1.softberry.com/berry.phtml). The *N*-myristoylation and *S*-palmitoylation sites of GuCPK were predicted by an updated software, CSS-Palm 2.0 [58].

Gene structure was carried out using the Gene structure display server (GSDS) server (http://gsds.cbi.pku.edu.cn/) by alignment of the ORF against *GmCPK* genomic sequences. Paralogues was acquired under multiple sequence alignment >70%. *Ka* and *Ks* values were calculated by DnaSP 5.0 software (Barcelona, Catalonia, Spain). The *Ka*/*Ks* ratios were used to assess the selection pressure; the ratio >1, =1, or <1 indicates positive, neutral, or negative selection, respectively [59]. The amino acid sequences of the duplicated GuCPK pairs were used to analyze the Tajima relative rate tests by MEGA 5.0 [60]. The syntenic relationships of paralogues and/or orthologues among *G. uralensis*, *Arabidopsis,* and *G. max* were identified using the Circos program.

### 4.3. Cis-Element Analysis in GuCPK Promoter Regions

The 1.5-kb sequence of the genomic sequence upstream to the start codon of each *GuCPK* was obtained; the *cis*-element distribution in *GuCPK* promoter regions was analyzed by the online tool PlantCARE server [61].

### 4.4. Plant Materials and Salt Treatment

Wild *G. uralensis* was used as the experimental material. The full granule seeds were treated with 98% concentrated sulfuric acid for 25 min to break the seed dormancy, with a subsequent rinse three times by sterilized distilled water and disinfection by 0.1% of HgCl for 10 min. The sterilized seeds were germinated on vermiculite culture in an automatic climate chamber (200 μmol m^−2^·s^−1^ light intensity, 16 h light/8 h dark photoperiod, 50–55% relative humidity, and 28 °C/25 °C day/night culture temperature). Roots, stems, and leaves were collected from 45-day-old *G. uralensis* seedlings. The 42-day-old *G. uralensis* seedlings were cultured for three days by hydroponics, and then were treated under different concentrations of salt stress.

Seedlings were removed out and subjected to the liquid mediums containing 0 mM, 15 mM, 30 mM, 45 mM, and 90 mM of NaCl, 0 mM, 2.5 mM, 5 mM, 10 mM, and 20 mM CaCl_2_, or different concentration combinations of NaCl and CaCl_2_, respectively. The culture medium was replaced every three days, and the root materials were collected after seven days of continuous stress. All the materials were frozen in liquid nitrogen immediately after collection and stored at −80 °C for further use. There were three independent replicates in each treatment, and 15 seedlings in each group.

### 4.5. Determination of Glycyrrhizic Acid and Flavonoids

*G. uralensis* root materials were washed three times with deionized water after seven days of stress treatment, and then were quickly put into an oven at 105 °C for 15 min, with subsequent baking at 70 °C to constant weight. The dried *G. uralensis* roots were ground into powder. An approximately 50-mg sample was extracted with supplementation of 2.5 mL of extraction solution (methanol: water: 36% glacial acetic acid = 71:28:1) in an ultrasonic crusher for 30 min. The sample mixture was filtered through a filter paper. The extraction procedure was repeated two times, and all the filtered extract solutions were collected and placed into a 10-mL centrifugal tube.

Accurate 3-mg standard monoammonium glycyrrhizinate (MAG) salt was dissolved in 3 mL of methanol to prepare the 1 mg/mL standard storage solution. Volumes of 25 μL, 50 μL, 100 μL, and 200 μL of MAG standard solution were added to 1 mL of extraction solution (methanol: water: 36% glacial acetic acid = 71:28:1), respectively. The peak time and area were determined by high-performance liquid chromatography (HPLC) (Agilent, Santa Clara, CA, USA) at 254 nM to produce the standard curve (Supplementary Figure S2). The glycyrrhizic acid content was carried out at 254 nM with the procedure of 5 μL of injection volume, 1 mL/min flow rate, and a 30 °C column temperature.

An accurate 3-mg standard Naringin was dissolved in 3 mL of chromatographic methanol to obtain the 1 mg/mL standard solution. Volumes of 100 μL, 200 μL, 400 μL, and 800 μL of Naringin standard solution were added to 1 mL of methanol and 0.5 mL of 10% KOH, respectively, and were then diluted with methanol to 10 mL with placement at room temperature for 5 min. The peak time and area were determined by HPLC at 416 nM to prepare the standard curve (Supplementary Figure S2). The 200-μL extract solution from different groups were pipetted into a 96-well plate to measure the total flavonoids at 416 nM.

### 4.6. Analysis the Expression Pattern of GuCPKs

The total RNA isolated from different *G.uralensis* tissues using a RNAprep pure plant kit (TIANGEN, Beijing, China) was used as a template to synthetize cDNA that was utilized for RT- and qRT-PCR analyses, with the specific primers designed based on the sequences of the 23 *GuCPKs* genes (Supplementary Table S1). *GuActin* (Accession no. GQ404511) was used as internal control. qRT-PCR was performed using 10-fold diluted cDNA and SruperReal PreMix Plus (SYBR Green) (TIANGEN, Beijing, China) on the 96-well plates in a LightCycler^®^ 480 Real-Time PCR System (Roche Diagnostics International, Rotkreuz, Switzerland). The Log_10_^2-^^ΔΔ^^Ct^ method was used for data processing, and the horizontal clustering of heat maps was analyzed by R language.

## Figures and Tables

**Table 1:** The *Ka*/*Ks* ratios for duplicate *CPK* genes in *G. uralensis*.

Paralogous Genes	*Ka* ^a^	*Ks* ^b^	*Ka*/*Ks*	Selective Pressure
*GuCPK1–GuCPK6*	0.0584	0.6518	0.0895	Purifying selection
*GuCPK4–GuCPK16*	0.0798	0.4052	0.1969	Purifying selection
*GuCPK8–GuCPK23*	0.0760	0.5474	0.1388	Purifying selection
*GuCPK13–GuCPK15*	0.0627	0.5245	0.1195	Purifying selection

^a^*Ka*: nonsynonymous substitution rate; ^b^*Ks*: synonymous substitution rate.

**Table 2:** Tajima relative rate tests of *CPK* gene pairs in *G. uralensis*^a^.

Testing Group	Mt ^b^	M1 ^c^	M2 ^d^	Χ^2^	*P* ^e^
*GuCPK4/GuCPK16* with *GmCPK6*	398	39	19	6.90	0.00864
*GuCPK1/GuCPK6* with *GmCPK25*	296	9	16	1.96	0.16151
*GuCPK1/GuCPK6* with *GmCPK26*	300	9	16	1.96	0.16151
*GuCPK8/GuCPK23* with *GmCPK23*	422	19	24	0.58	0.44577
*GuCPK13/GuCPK15* with *GmCPK9*	426	32	11	10.26	0.00136

^a^ The Tajima relative rate test was used to examine the equality of evolutionary rate between *G. uralensis* paralogues; ^b^ Mt is the sum of the identical sites in all three sequences tested; ^c^ M1 is the number of unique differences in the first paralog; ^d^ M2 is the number of unique differences in the second paralog; ^e^ If *p* < 0.05, the test rejects the equal substitution rates between the two duplicates and infers that one of the two duplicates has an accelerated evolutionary rate.

## Supplementary Materials

Supplementary materials can be found at https://www.mdpi.com/1420-3049/24/9/1837/s1. Figure S1: Expression profiles of the 23 *GuCPK* genes in *G. uralensis* roots under CaCl_2_ and NaCl stimulations. Figure S2: Standard curves of glycyrrhizic acid and flavonoids determinations. Table S1: Primers used in this work. Table S2: Sequences of nucleic acid and protein of the 23 *GuCPK* genes. Table S3: Detailed information of *GuCPKs*. Table S4: Putative *cis*-acting elements in promoter regions of *GuCPK* genes.

## Abbreviations

aa	Amino acid
ABA	Abscisic acid
ABRE	ABA-responsive element
ARE	Anaerobic induction
CPK	Calcium-dependent protein kinases
DLK	Dual leucine-zipper kinase
EF	Effective factor
ERF	Ethylene-responsive element
GA	Gibberellin
GARE	Gibberellin response element
GSDS	Gene structure display server
HMM	Hidden Markov model
HPLC	High-performance liquid chromatography
LTR	Low-temperature responsiveness
MAG	Monoammonium glycyrrhizinate
MBSI	MYB binding site involving in flavonoid biosynthetic gene regulation
MEME	Multiple expectation-maximization (Em) for motif elicitation
MRE	Mineralocorticoid response element
MYB	v-myb avian myeloblastosis viral oncogene homolog
MW	Molecular weight
ORF	Open reading frame
*p*I	Isoelectric point
Ser	Serine
Thr	Threonine
