# Supplementary material for: Calcium-Dependent Protein Kinase Genes in Glycyrrhiza Uralensis Appear to be Involved in Promoting the Biosynthesis of Glycyrrhizic Acid and Flavonoids under Salt Stress
Source: Molecules. 2019 May 13;24(9):1837. doi: 10.3390/molecules24091837 (PMC6539831; doi:10.3390/molecules24091837)
Supplement: Supplementary file 1 [file molecules-24-01837-s001.zip › molecules-493997-supple-proofed/Figure S1.pdf]

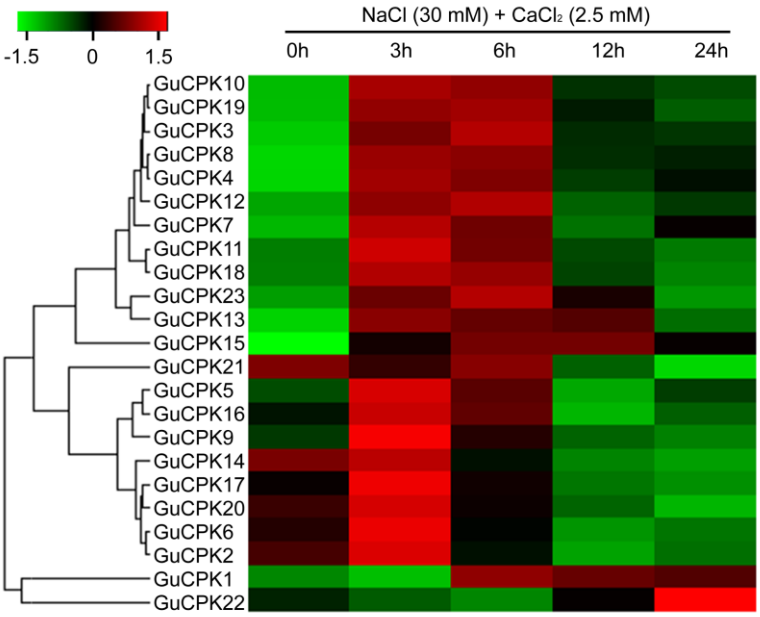

Figure S1. Expression profiles of the 23 *GuCPK* genes in *Glycyrrhiza uralensis* roots under $CaCl_2$ and NaCl stimulations. The heatmap of the 23 *GuCPKs* at 0, 3, 6, 12 and 24 h treatment of the mixture of 2.5 mM $CaCl_2$ and 30 mM NaCl was performed by R package based on the qRT-PCR data. Each value indicates the mean of the expression levels of three independent replicates. Genes that highly or weakly expressed in different treated materials were colored by red or green, respectively. The color bar was showed at the upper apex.
