# Supplementary material for: Calcium-Dependent Protein Kinase Genes in Glycyrrhiza Uralensis Appear to be Involved in Promoting the Biosynthesis of Glycyrrhizic Acid and Flavonoids under Salt Stress
Source: Molecules. 2019 May 13;24(9):1837. doi: 10.3390/molecules24091837 (PMC6539831; doi:10.3390/molecules24091837)
Supplement: Supplementary file 1 [file molecules-24-01837-s001.zip › molecules-493997-supple-proofed/Figure S2-R1.pdf]

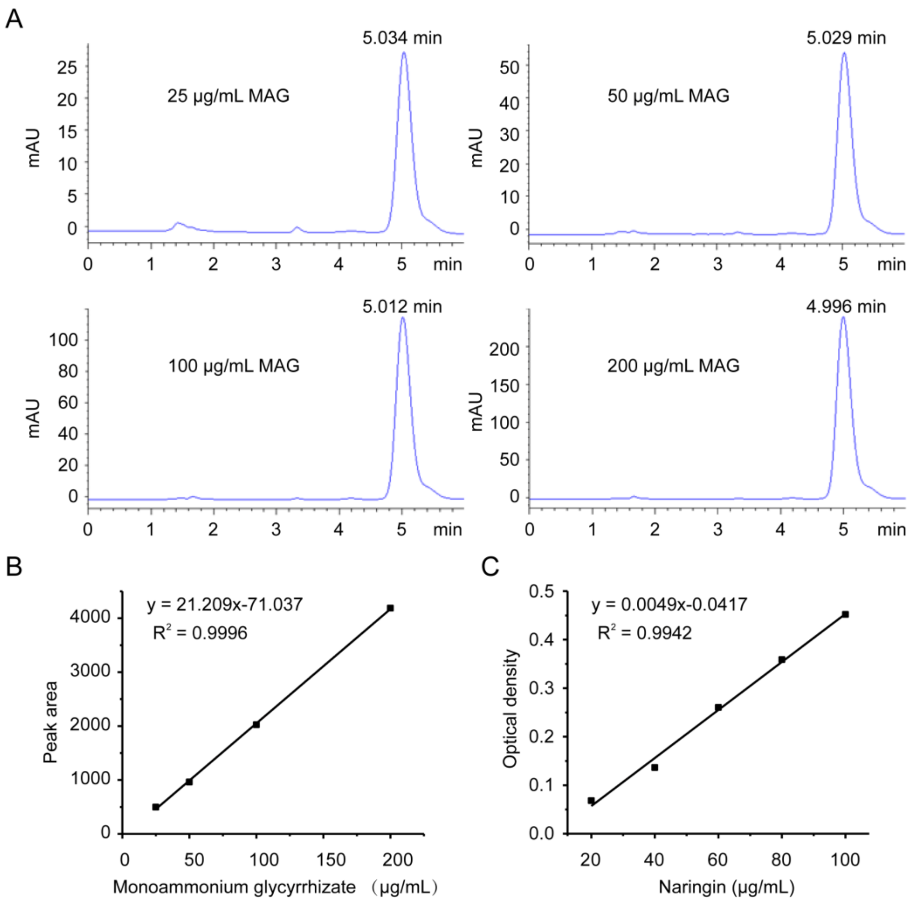

Figure S2. Standard curves of glycyrrhizic acid and flavonoids determinations. (A) HPLC peak area of monoammonium glycyrrhizate. Retention time is marked on the peak. (B) Standard curve for glycyrrhizic acid determination. Monoammonium glycyrrhizate concentrations in the linear range from 25 to 200 μg/mL are displayed. (C) Standard curve for flavonoids determination. Naringin concentrations in the linear range from 20 to 100 μg/mL are presented.
