# Supplementary material for: Calcium-Dependent Protein Kinase Genes in Glycyrrhiza Uralensis Appear to be Involved in Promoting the Biosynthesis of Glycyrrhizic Acid and Flavonoids under Salt Stress
Source: Molecules. 2019 May 13;24(9):1837. doi: 10.3390/molecules24091837 (PMC6539831; doi:10.3390/molecules24091837)
Supplement: Supplementary file 1 [file molecules-24-01837-s001.zip › molecules-493997-supple-proofed/Supplementary Table S1.pdf]

Supplementary Table S1. Primers used in this work.

Primer name	Primer sequence (5'-3')	Product size (bp)
GuCPK1-sense	GAGAAAGATGGGAAATGACG	280
GuCPK1-antisense	TTCAGGCTGAGACTGGTAAA	
GuCPK2-sense	GCAACCTACGAGGACAACGA	195
GuCPK2-antisense	ATAGTAAGGACTCCCAACAATCT	
GuCPK2F-sense*	ATGGGAAACTGCAACGT	1653
GuCPK2F-antisense*	TCAAACCACTACCGCTT	
GuCPK3-sense	GGTGGGCAGTGCTTACTATGT	152
GuCPK3-antisense	TGGCGTTAAATATTCCCTTTTCAGT	
GuCPK4-sense	CGCATTACTTCTGCTCAGGT	187
GuCPK4-antisense	TTGCCTTCAAACCTTGGATC	
GuCPK5-sense	CACTCATTCTGATCCCTCCG	155
GuCPK5-antisense	CATAATACGCTTGGTTGTCG	
GuCPK6-sense	ATGACGATGCCGTTCACCTC	289
GuCPK6-antisense	ATGACTCCAGCACTCCAGAT	
GuCPK6F-sense*	ATGGGAAATTGCTGTACG	1568
GuCPK6F-antisense*	TCATCTACCTTCATTTTCTAA	
GuCPK7-sense	ACGCAGCCAATCGCAAAC	283
GuCPK7-antisense	TGTCCGTGACCCAACAAC	
GuCPK8-sense	CCTCAGCAACGACGAGAATT	107
GuCPK8-antisense	CATAATAGGCACTCCCAACAA	
GuCPK9-sense	GCAGGGCTTCAAGCAGAGTC	169
GuCPK9-antisense	CCAGTTTCACCTTTGGGATG	
GuCPK9F-sense*	CTTAAGGCAATAGATTTTGGAATGTCT	370
GuCPK9F-antisense*	GCAACACCGTCAACCTGAATCCAAG	
GuCPK10-sense	CCATCGCCGAGGTTGTTAGG	270
GuCPK10-antisense	CAGTCTCAGCCCAAAAAGGA	
GuCPK10F-sense*	ATGGGGAATTGCTGCACGT	1650
GuCPK10F-antisense*	TCAAACCACAACAGCTTGGCCACTG	
GuCPK11-sense	ATAGGCGGGTTGAAAGAGTT	144
GuCPK11-antisense	ATCAGCCGCATCCATAAGAG	
GuCPK12-sense	AACTAATGAGCCTGAATCCC	288
GuCPK12-antisense	TACTGGTGTCCCGTTGCTTT	
GuCPK13-sense	GTTGTCAGCCTTCTCCTATT	257
GuCPK13-antisense	CATTGCCTACTATTCCCAGA	
GuCPK13F-sense*	ATGGCGAAATCGAATTCGAGC	1615
GuCPK13F-antisense*	TCAATTTTCATGCAATGAATCATCT	
GuCPK14-sense	CCACAAGAACACTGGGAAAC	194
GuCPK14-antisense	CTGCACACAATTCCATCACC	
GuCPK15-sense	TTTTATCACGCCTGAAGCAA	265
GuCPK15-antisense	TCACCATAGTCAAGTGTCCC	
GuCPK16-sense	ATGGGTGGCTGTCTGAGTAA	115

GuCPK16-antisense	TTGATTGGTTCACAAAGGGT	
GuCPK16F-sense*	AAATCTCTCTTTTTTTGTGCCA	1705
GuCPK16F-antisense*	AAACTTTTAGTGAGCCCTTCCC	
GuCPK17-sense	GCTGTTGCTGTCCATGTTGT	221
GuCPK17-antisense	GCCTAGCTGCCTTTCTCTCA	
GuCPK17F-sense*	GATGCTTATAGTGTTAGATGGG	1756
GuCPK17F-antisense*	CTTAACATACAGGTAGTGCCTC	
GuCPK18-sense	CTATGTGGCTCCAGAGGTGT	247
GuCPK18-antisense	GGTAATGCGTTTCTTAGGGT	
GuCPK19-sense	GGCAAATGTTAGAACCAGACC	109
GuCPK19-antisense	ACATCTCCAAGAGGAACATTAGG	
GuCPK20-sense	TTTTGGGATGAAACGGAACA	196
GuCPK20-antisense	GAGCAACACCTCCAACCTGA	
GuCPK21-sense	TGGGCAGAATCCGAACATGGTA	158
GuCPK21-antisense	AAAACTTCCCCCGCCGTCAG	
GuCPK22-sense	GTTCTTAGAGTGGTTGCCGATA	168
GuCPK22-antisense	CTGGACATCAGGATCAGGAATA	
GuCPK22F-sense*	ACCTTGTCATGGAGCTCTGC	792
GuCPK22F-antisense*	ATCAGGAATAGCGTGTCCAATC	
GuCPK23-sense	TCTCCCTAAACTGGGTACTAAA	186
GuCPK23-antisense	CCGCTCTTATCCTGGTCAAA	
GuActin-sense	CCTCAACCCAAAGGTCAACAG	
GuActin-antisense	GACCAGCGAGATCCAAACGAA	

*: Primers used for amplifying the full-length open reading frame sequences of *GuCPKs*.
