# Supplementary material for: Calcium-Dependent Protein Kinase Genes in Glycyrrhiza Uralensis Appear to be Involved in Promoting the Biosynthesis of Glycyrrhizic Acid and Flavonoids under Salt Stress
Source: Molecules. 2019 May 13;24(9):1837. doi: 10.3390/molecules24091837 (PMC6539831; doi:10.3390/molecules24091837)
Supplement: Supplementary file 1 [file molecules-24-01837-s001.zip › molecules-493997-supple-proofed/Supplementary Table S3-R1.pdf]

Supplementary Table S3. Detailed information of *GuCPKs*.

Gene name	Scaffold location[a]	Predicted Protein[b]				N-termianal AA.	Palmitoylation sites[c]	Myristoylation sites[d]	Subcellular location[e]
		ORF (bp)	Length (aa)	MW (kDa)	*p*I				
GuCPK1	scaffold0019 (-): 599558 - 604247	1644	547	61.63	6.11	MGNCCATP	Yes	n.d.	P
GuCPK2	scaffold0020 (-): 183531 - 187574	1542	513	57.69	5.86	MGNCNVCA	Yes	n.d.	P
GuCPK3	scaffold0026 (+): 234895 - 240067	1647	548	61.06	5.95	MGCHGSKE	Yes	Yes	P
GuCPK4	scaffold0029 (-): 62755 - 67593	1638	545	61.45	6.31	MGSCLSLG	Yes	Yes	P
GuCPK5	scaffold0059 (+): 59588 - 69104	1683	560	62.78	5.79	MGNTCRGS	Yes	n.d.	P
GuCPK6	scaffold0110 (+): 167069 - 171335	1503	500	56.99	6.22	MGNCCTTP	Yes	Yes	P
GuCPK7	scaffold0140 (+): 307174 - 312863	1701	566	64.30	9.22	MGLCFSST	Yes	Yes	P
GuCPK8	scaffold0143 (+): 95483 - 99940	1545	514	58.11	6.60	MGNRISRK	n.d.	Yes	P
GuCPK9	scaffold0170 (-): 142051 - 146381	2025	674	75.08	6.12	MGNNCVGS	Yes	n.d.	P
GuCPK10	scaffold0204 (+): 73080 - 77284	1626	541	61.34	5.85	MGNCCTCV	Yes	Yes	P
GuCPK11	scaffold0217 (+): 42799 - 46457	1620	539	60.92	5.76	LEREREGE	n.d.	n.d.	P
GuCPK12	scaffold0224 (-): 38473 - 43745	1731	576	64.57	5.34	MGNTCVGP	Yes	n.d.	P
GuCPK13	scaffold0252 (+): 113646 - 118430	1650	549	62.01	5.18	MAKSNSST	n.d.	n.d.	P
GuCPK14	scaffold0254 (+): 139702 - 143097	1593	530	59.18	5.66	MGNCCSGG	Yes	Yes	P
GuCPK15	scaffold0282 (-): 102302 - 107315	1482	493	55.77	5.33	MSKSNTVV	n.d.	n.d.	P
GuCPK16	scaffold0364 (-): 96991 - 102305	1692	563	63.57	8.51	MGGCLSKK	Yes	Yes	P
GuCPK17	scaffold0475 (+): 46323 - 51388	1716	571	63.15	5.73	MGNNCVGS	Yes	n.d.	P
GuCPK18	scaffold0702 (-): 63458 - 70368	1581	526	59.05	5.47	MGLGVFKA	n.d.	n.d.	P
GuCPK19	Scaffold1037 (+): 77874 - 84236	1620	539	60.29	6.08	MGNCCRSP	Yes	n.d.	P
GuCPK20	Scaffold1288 (+): 66653 - 71753	1824	607	66.40	5.51	MGNNWVGP	n.d.	n.d.	P
GuCPK21	Scaffold1585 (+): 27089 - 31953	2097	698	78.45	5.28	MGNCCSQG	Yes	Yes	P
GuCPK22	Scaffold1732 (+): 21924 - 25798	1560	519	58.83	5.63	MGSCVSTQ	Yes	Yes	P
GuCPK23	Scaffold2052 (-): 7400 - 13113	1542	513	57.64	6.01	MGNCSSGG	Yes	Yes	P

[a] scaffold location: [Scaffold number (Orientation): start - end], '+' and '-' indicate the forward and reverse orientation, respectively.

[b] aa, amino acid; MW, molecular weight; *p*I, isoelectric point.

[c] The predicted palmitoylation site of each GuCPK was using CSS-Palm 4.0.

[d] The predicted palmitoylation site of each GuCPK was using Myristoylator..

[e] The predicted subcellular location of each GuCPK was analyzed using Softberry server.
