# Supplementary material for: Calcium-Dependent Protein Kinase Genes in Glycyrrhiza Uralensis Appear to be Involved in Promoting the Biosynthesis of Glycyrrhizic Acid and Flavonoids under Salt Stress
Source: Molecules. 2019 May 13;24(9):1837. doi: 10.3390/molecules24091837 (PMC6539831; doi:10.3390/molecules24091837)
Supplement: Supplementary file 1 [file molecules-24-01837-s001.zip › molecules-493997-supple-proofed/Supplementary Table S4.pdf]

Supplementary Table S4. Putative *cis*-acting element sequences in promoter regions of *GuCPK* genes.

Classification	Symbol	*cis*-element	Sequence*	Description
Transcription initiation	T	TATA box	TAATA	core promoter element around -30 of transcription start
	C	CAAT box	CAATT	common *cis*-acting element in promoter and enhancer regions
Phytohormone responsiveness	A	ABRE	ACGTGGC	*cis*-acting element involved in the abscisic acid responsiveness
	E	ERE	ATTTCAAA	Ethylene-responsive element
	N	TGA	AACGAC	auxin-responsive element
	G	GARE	WAACAR	Gibberellin-responsive element
	P	P-box	CCTTTTG	Gibberellin-responsive element
Stress responsiveness	M	MBS	TAACTG	MYB binding site involved in drought-inducibility
	S	MBSI	aaaAaaCSGTTA	MYB binding site involved in flavonoid biosynthetic genes regulation
	R	MRE	AACCTAA	MYB binding site involved in light responsiveness
	H	HSE	AAAAAATTTC	*cis*-acting element involved in heat stress responsiveness
	D	TC-rich	ATTTTCTCCA	*cis*-acting element involved in defense and stress responsiveness
	J	G-box	CACGTT	*cis*-acting regulatory element involved in light responsiveness

	B	Box I	TTTCAAA	light responsive element
	O	ARE	TGGTTT	*cis*-acting regulatory element essential for the anaerobic induction
	F	AE-box	AGAAACAA	part of a module for light response
	I	I-box	GATAAGATT	part of a light responsive element
	P	3-AF1	AAGAGATATTT	light responsive element
	L	LTR	CCGAAA	*cis*-acting element involved in low-temperature responsiveness
High transcription	U	5'UTR Py-rich stretch	TTTCTTCTCT	*cis*-acting element conferring high transcription levels

*: N = A, C, G or T; S = C or G.
