# Supplementary figures and images for: Calcium-Dependent Protein Kinase Genes in Glycyrrhiza Uralensis Appear to be Involved in Promoting the Biosynthesis of Glycyrrhizic Acid and Flavonoids under Salt Stress
Source: Molecules. 2019 May 13;24(9):1837. doi: 10.3390/molecules24091837 (PMC6539831; doi:10.3390/molecules24091837)

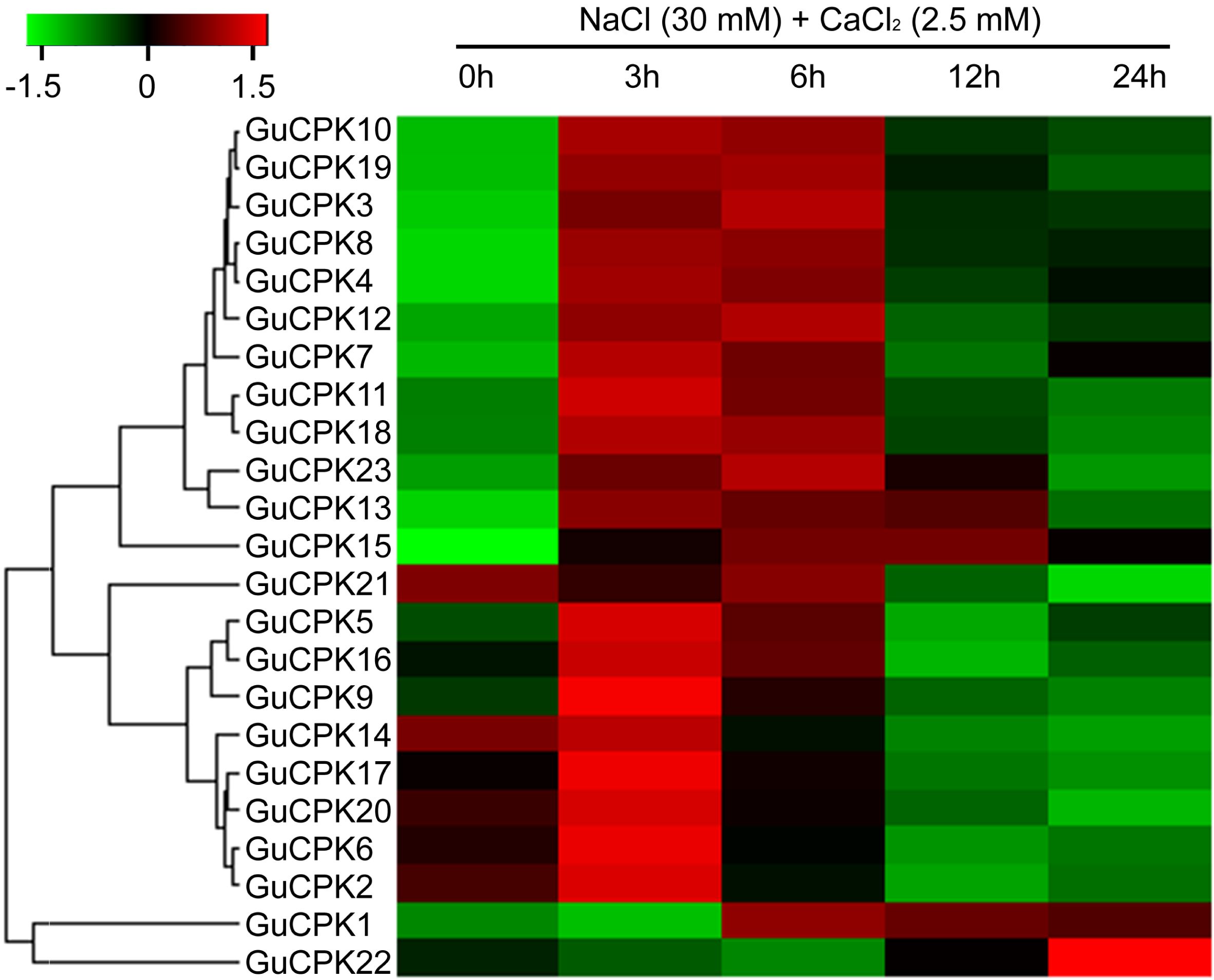

Supplement: Supplementary file 1 [file molecules-24-01837-s001.zip › molecules-493997-supple-proofed/Figure S1.tif]

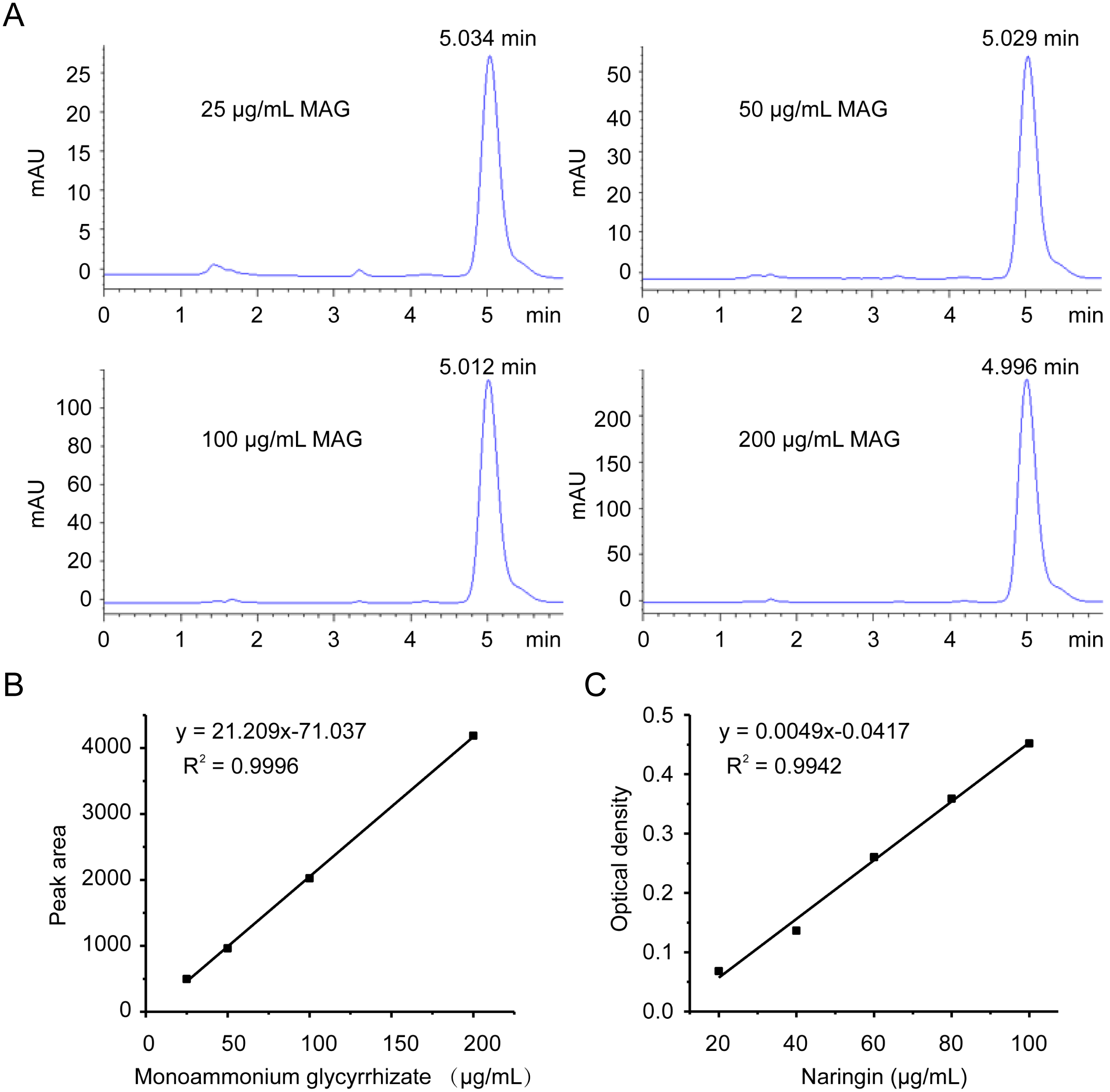

Supplement: Supplementary file 1 [file molecules-24-01837-s001.zip › molecules-493997-supple-proofed/Figure S2-R1.tif]
